# Hub genes identification and validation of ferroptosis in SARS-CoV-2 induced ARDS: perspective from transcriptome analysis

**DOI:** 10.3389/fimmu.2024.1407924

**Published:** 2024-08-07

**Authors:** Yutang Li, Li Tang, Fang Wang, Chencheng Gao, Qi Yang, Liyu Luo, Jiahang Wei, Qiuyun Tang, Mingran Qi

**Affiliations:** ^1^ Department of Pathogen Biology, College of Basic Medical Sciences, Jilin University, Changchun, China; ^2^ The First Hospital of Jilin University, Jilin University, Changchun, China; ^3^ The Second Hospital of Jilin University, Jilin University, Changchun, China; ^4^ College of Sports Medicine and Physical Therapy, Beijing Sport University, Beijing, China; ^5^ Department of Oncology, Health Center of Chicheng Town, Suining, China

**Keywords:** ARDS, SARS-CoV-2, ferroptosis, WGCNA, bioinformation

## Abstract

**Introduction:**

Acute Respiratory Distress Syndrome (ARDS) poses a significant health challenge due to its high incidence and mortality rates. The emergence of SARS-CoV-2 has added complexity, with evidence suggesting a correlation between COVID-19 induced ARDS and post-COVID symptoms. Understanding the underlying mechanisms of ARDS in COVID-19 patients is crucial for effective clinical treatment.

**Method:**

To investigate the potential role of ferroptosis in SARS-CoV-2 induced ARDS, we conducted a comprehensive analysis using bioinformatics methods. Datasets from the Gene Expression Omnibus (GEO) were utilized, focusing on COVID-19 patients with varying ARDS severity. We employed weighted gene co-expression network analysis (WGCNA), differential gene expression analysis, and single-cell sequencing to identify key genes associated with ferroptosis in ARDS. Hub genes were validated using additional GEO datasets and cell experiment.

**Result:**

The analysis discerned 916 differentially expressed genes in moderate/severe ARDS patients compared to non-critical individuals. Weighted Gene Co-expression Network Analysis (WGCNA) unveiled two modules that exhibited a positive correlation with ARDS, subsequently leading to the identification of 15 hub genes associated with ferroptosis. Among the noteworthy hub genes were MTF1, SAT1, and TXN. Protein-protein interaction analysis, and pathway analysis further elucidated their roles. Immune infiltrating analysis highlighted associations between hub genes and immune cells. Validation in additional datasets confirmed the upregulation of MTF1, SAT1, and TXN in SARS-CoV-2-induced ARDS. This was also demonstrated by qRT-PCR results in the BEAS-2B cells vitro model, suggesting their potential as diagnostic indicators.

**Discussion:**

This study identifies MTF1, SAT1, and TXN as hub genes associated with ferroptosis in SARS-CoV-2-induced ARDS. These findings provide novel insights into the molecular mechanisms underlying ARDS in COVID-19 patients and offer potential targets for immune therapy and targeted treatment. Further experimental validation is warranted to solidify these findings and explore therapeutic interventions for ARDS in the context of COVID-19.

## 1 Introduction

Acute respiratory distress syndrome (ARDS) is an acute, diffuse, inflammatory lung injury, which can be caused by a range of risk factors such as pneumonia, non-pulmonary infection, trauma, transfusion, burn, aspiration, or shock. The clinical hallmarks of ARDS are arterial hypoxemia and diffuse radiographic opacities associated with increased shunting, increased alveolar dead space, and decreased lung compliance, which caused by pulmonary vascular and epithelial permeability, lung edema, and gravity-dependent atelectasis (1). The incidence rate and mortality rates of ARDS is both high, in a randomized controlled clinical trial, the mortality rate in moderate and severe ARDS patients exceeded 40% (2). Therefore, ARDS causes great health damage to patients and poses challenges for clinical treatment and care.

Since the discovery of SARS-CoV-2 in 2019, the virus has rapidly spread worldwide, causing severe global public health issues. It is worth noting that SARS-CoV-2 has the ability to inflict damage upon tracheal epithelial cells and lung alveoli, triggering an inflammatory cytokine storm, which can subsequently lead to the development of ARDS (3). What is even more concerning is that there is evidence suggesting that some ARDS survivors may experience severe and long-lasting complications and sequelae, including anosmia, cognitive impairments, breathlessness, poor exercise tolerance, arrhythmias, myalgias, depression, and fatigue (4).

Currently, some reviews (5–8) suggests a close relationship between the severity of “POST COVID-19” symptoms and the high viral load, prolonged inflammation during COVID-19 infection, and the individual’s immune environment. A retrospective analysis found that severe cases of COVID-19 infection are more prone to experiencing “POST COVID-19” symptoms, accompanied by excessive activation of innate immune cells, a decrease in Naive T and B cells (9), mast cell activation can also induce inflammation in COVID-19 and lead to “POST COVID-19” symptoms (10), moreover, single-cell RNA-seq data from moderate to severe SARS-CoV-2-infected patients show a decrease in lymphocytes in some cases (11). In addition, the dysregulation of CD8+ T cell response is associated with impaired lung function after acute coronavirus disease (12). Therefore, investigating the mechanisms underlying the development of moderate and severe ARDS in SARS-CoV-2 is of great significance importance for clinical treatment and disease recovery.

In 2012, Dixon et al. (13) proposed that ferroptosis is a regulated cell death process caused by excessive lethal lipid peroxidation and those cells dying by ferroptosis primarily exhibit shrunken and damaged mitochondria by electron microscopy, with few other morphological changes evident prior to the point of cell death (14). The occurrence of ferroptosis involves both endogenous and exogenous pathways. The endogenous pathway involves the inhibition of cell membrane transport proteins, such as the cystine/glutamate transporter (system Xc-) or the activation of iron transport proteins, transferrin, and lactotransferrin. The exogenous pathway involves the inhibition of intracellular antioxidant enzymes, such as glutathione peroxidase (GPX4). Both can induce iron-dependent lipid peroxidation, leading to structural and functional damage of biological membranes, ultimately resulting in cell death (15).

Ferroptosis is closely associated with various diseases and pathological processes, such as infection, inflammation (16), autoimmune diseases, acute kidney injury (17) and so on. Inflammatory mediators can induce the generation of reactive oxygen species (ROS), disrupting the balance between oxidation and antioxidation, leading to localized oxidative stress and inducing ferroptosis (18, 19). Meanwhile, ferroptosis can lead to immune cell accumulation, promote the release of inflammatory cytokines, and exacerbate ARDS. Therefore, ferroptosis and inflammation form a self-amplified loop, which further promotes organ damage (20).

Some experiments have shown that the occurrence of ferroptosis is closely related to ARDS. The ferroptosis inhibitor ferrostatin-1 (Fer-1) can alleviate lipopolysaccharide-induced acute lung injury (21). The inhibitor of apoptosis-stimulating protein of p53 (iASPP) can significantly ameliorate ARDS caused by intestinal ischemia-reperfusion (22). Moreover, miR-125b-5p in adipose-derived stem cells exosome can alleviate pulmonary microvascular endothelial cell ferroptosis in sepsis lung injury by increasing the expression of Nrf2 and GPX4 (23). Recent studies have proven that activating the α7 nicotinic acetylcholine receptor (α7nAchR) in lung tissue (24) or blocking mTOR signaling (25) can significantly ameliorate the sepsis-induced ARDS by inhibiting ferroptosis. It suggests that there may still be numerous undiscovered pathways and mechanisms associated with the development of ferroptosis in ARDS.

However, limited information is currently available regarding the potential role of ferroptosis in the pathogenesis of virus-induced ARDS, (such as influenza and SARS-CoV-2). Therefore, there is an urgent need to investigate the role and underlying mechanisms of ferroptosis in virus-induced ARDS.

## 2 Materials and methods

### 2.1 Data download and processing

Download datasets GSE172114, GSE157103, GSE163426, and GSE149689 from the Gene Expression Omnibus (GEO) database (https://www.ncbi.nlm.nih.gov/geo). The detail information is shown in **Table 1**. The whole blood RNA samples in GSE172114 were obtained from patients hospitalized in France during the first wave of the COVID-19 pandemic (March to April 2020) in the northern region of Alsace, who were infected with SARS-CoV-2. Patients were classified into two groups according to the Berlin definition (26): (i) 46 ICU critically ill patients with moderate or severe ARDS, and (ii) 23 non-critically ill patients (27). In GSE157103, we selected whole blood total RNA samples from patients with COVID-19 and divided them into two groups: (i) patients requiring mechanical ventilation (n=42), and (ii) patients not requiring mechanical ventilation (n=58). GSE163426 extracted total RNA from tracheal aspirates of patients with COVID-19 induced ARDS (n=15) and mechanically ventilated patients without evidence of pulmonary disease (n=5). The single-cell sequencing data in GSE149689 came from healthy donors (n=4) and patients with COVID-19 of varying clinical severity, including severe, mild, and asymptomatic cases (n=11) from Asan Medical Center, Severance Hospital, and Chungbuk National University Hospital (28).

**Table 1:** The information of GEO series.

	Matrix	Country	Pathogen	Sample	Experiment group	Control group
Derivation cohorts	GSE172114	Alsace (France)	COVID-19	Total RNA of total blood	46 critical (ARDS), ICU	23 non-critical (Control), non-critical care ward
Validation cohorts	GSE157103	Wisconsin (USA)	COVID-19	Total RNA of leukocytes	42 critical (ARDS), ICU	58 non-critical (Control), non-critical care ward
	GSE163426	California (USA)	COVID-19	Total RNA of tracheal aspirate	15 critical (ARDS), ICU	5 non-critical (Control), non-critical care ward
	GSE149689	Daejeon (Korea)	COVID-19	Single-cell RNA sequencing	severe, mild, and asymptomatic (n=11), healthy donors (n=4)	

### 2.2 Validation in the GEO dataset

We chose GSE157103 and GSE163426 for validation, and visualized the distribution of hub genes in the aforementioned matrices through violin plots to explore whether the expression trends of hub genes in these matrices are consistent. We defined the criteria for selecting the ultimate key genes closely associated with the Ferroptosis pathway in PPI and GO/KEGG analysis.

### 2.3 Differential gene expression analysis

Firstly, we downloaded the GSE172114 file from the GEO website and found that the data in the GSE172114 file had already been TMM normalized with edgeR and log2-transformed. Secondly, we performed differential gene expression analysis using the “limma” R package in R software (version 4.3.2) (29). We find differential expression genes with the screening criterion are p-value ≤ 0.05 and |logFC| ≥ 1. The “ggplot2” and “ComplexHeatmap” R package was used to draw volcano plots and heatmaps to display the differential expression of genes.

### 2.4 Co-expression network construction and consensus module detection

To seek genes that are more significantly associated with the COVID-19 induced ARDS, we utilized the WGCNA package in R to process the GSE172114 file. All co-expression networks were constructed using the BiocManager WGCNA package (30). (1) Data loading and preprocessing were conducted, including reverting the data back to pre-log2 transformation values, checking for missing values, and filtering out genes with expression levels below the threshold. The filtering criterion was set such that the values, when reverted to pre-log2 transformation, remained less than 0.5. (2) The pick SoftThreshold function of WGCNA was used to compute the scale-free topology fit index for a range of candidate powers from 1 to 30. This process helped determine the appropriate soft-thresholding power for constructing each module. If the index value for the reference dataset exceeded 0.85, the suitable power was identified. (3) Generation of the adjacency matrix and topological overlap matrix was accomplished, and through the use of the hclust function with “average” as the clustering method, we obtained the consensus modules, each containing a minimum of 80 genes. Then, the merge function within the WGCNA package was utilized to combine smaller modules with high similarity. The gene significance (GS) and module membership (MM) were used to identify the gene of high group significance and module membership in the modules. The significant module for ARDS was identified if: |GS|≥0.4 and |MM|≥0.6. The relationship between MM and GS in the module was statistically significant (p < 0.05).

### 2.5 Identification of hub genes related to ferroptosis

We downloaded ferroptosis-related genes from the FerrDb database (http://www.zhounan.org/ferrdb/current/, accessed on 13 November 2023), including driver, suppressor, marker and unclassified genes. We then took the intersection of these ferroptosis-related genes with the differential expression genes generated from limma and ARDS-related genes generated from WGCNA. The overlapping genes were visualized using a Venn diagram. In the STRING database, the species was set to Homo sapiens, with a minimum required interaction score of 0.350. Hub genes were visualized within the Protein-Protein Interaction (PPI) network.

### 2.6 Enrichment analysis

To further visualize the biological function of key genes related to ferroptosis and ARDS, Gene Ontology (GO) and Kyoto Encyclopedia of Genes and Genomes (KEGG) enrichment analyses were performed using the website (http://www.bioinformatics.com.cn/). The p-value of less than 0.05 was identified as a significant term.

### 2.7 Immune infiltrating

CIBERSORT is a method for characterizing cell composition of complex tissues from their gene expression profiles (31). LM22 gene signature files provided by CIBERSORT were used to estimate the abundances of immunocytes accompanying 1,000 permutations. In addition, the Wilcoxon test was applied to establish the differentially infiltrated immune cells in ARDS patients compared to non-critical patients. The correlation between hub genes and immune infiltrating cells was analyzed by Spearman’s rank correlation analysis and the results were visualized by the lollipop chart.

### 2.8 Single-cell sequencing analysis

The data were first processed using the Seurat package (32), and filtered according to the criteria of nFeature_RNA > 500 and percent.mt < 5. The FindClusters function was chosen with a resolution set at 1.0 for optimizing clustering modules. Each cluster and its spatial associations were identified through the Uniform Manifold Approximation and Projection (UMAP) method. Using the logfc.threshold option of FindAllMarkers, significance expressed genes in each cluster were selected based on the criteria of |avg_log2FC| > 0.5 and p_val_adj < 0.05. Subsequently, cell annotations for each cluster in the UMAP were performed through the CellMarker website, and key cellular biomarkers and selected hub genes from the sample were marked on the UMAP plot.

### 2.9 Establishment and validation of BEAS-2B cells in *Vitro* model

We established control and ARDS models using BEAS-2B cell (Cell Line Bank, Shanghai) stimulated with the S protein (Wuhan Jonk Biological technology Co.,Ltd, Wuhan). The control group was stimulated with 2 ml of 0.2 µg/ml S protein for 48 hours, while the ARDS group was stimulated with 2 ml of 5.0 µg/ml S protein for 48 hours. This experiment was approved and supported by the Basic Medical Sciences School of Jilin University. After 24 hours of stimulation, RNA was extracted using TRNzol Universal RNA Reagent (TIANGEN BIOTECH Co.,Ltd, Beijing), and cDNA was synthesized using All-In-One 5X RT MasterMix (Applied Biological Materials Inc., Canada). Quantitative Real-time PCR (qRT-PCR) was then conducted to measure the expression levels of SFTPD, SAT1, MTF1, and TXN. The expression of SFTPD was significantly down-regulated in the lung epithelial cells of COVID-19 induced ARDS patients (33). Primer sequences (Sangon Biotech Co., Ltd, Shanghai) of the four genes were described in **Supplementary Table 1**. Statistical analysis was performed using the t-test to compare the expression levels of SFTPD, SAT1, MTF1, and TXN between the groups stimulated with 0.2 µg/ml and 5.0 µg/ml S protein. Any p-value of less than 0.05 was considered statistically significant.

### 2.10 Statistical analysis

All statistical analyses were performed using R Studio (R Version 4.3.2) and GraphPad Prism 9.5.1 software (GraphPad Software, San Diego, CA, USA). The ANOVA and T-test was used in qRT-PCR analysis. Any P-value <0.05 was considered statistically significant.

## 3 Results

### 3.1 Co-expression network construction

**Figure 1:**
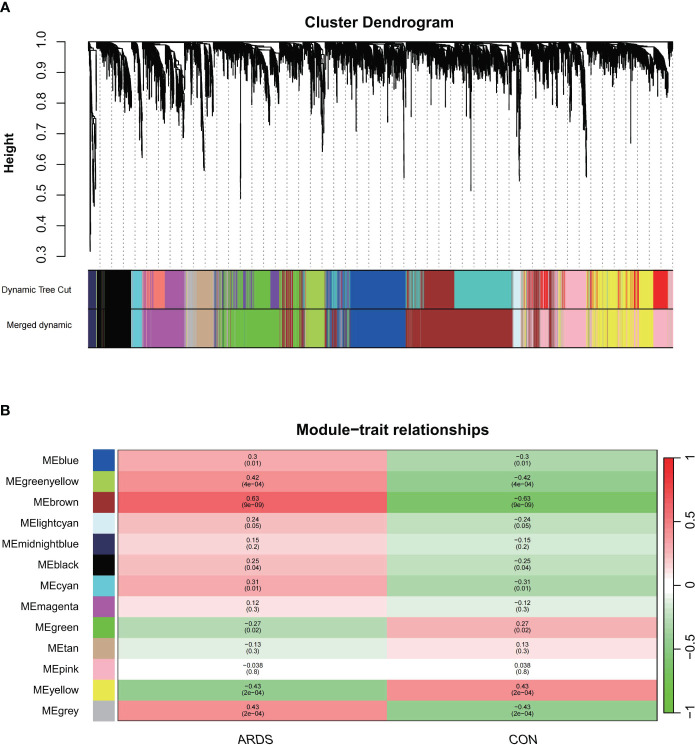
The construction of weighted gene co-expression networks. **(A)** Network TOM heatmap plot, **(B)** Modul-trait relationship.

We first sought to identify gene co-expression modules that we could subsequently link to the presence of ARDS. To do this, we utilized the hclust function within the WGCNA package with the average clustering method, we clustered the samples and generated a dendrogram of the sample clustering results. No outliers were observed at a cutting height less than 50,000. Using the one-step network construction function of the WGCNA R package and setting the cutting height to 0.25, smaller modules with high similarity were merged, resulting in the construction of 13 gene co-expression modules (**Figure 1A**). We selected a power β value that exhibited scale independence close to 1 and higher mean connectivity. The power β value was determined to be 22 when the fitting index reached 0.85, and the mean connectivity was at its maximum. (**Supplementary Figures S1A**, **B**).

### 3.2 Identification of the clinically significant modules and key genes

**Figure 2:**
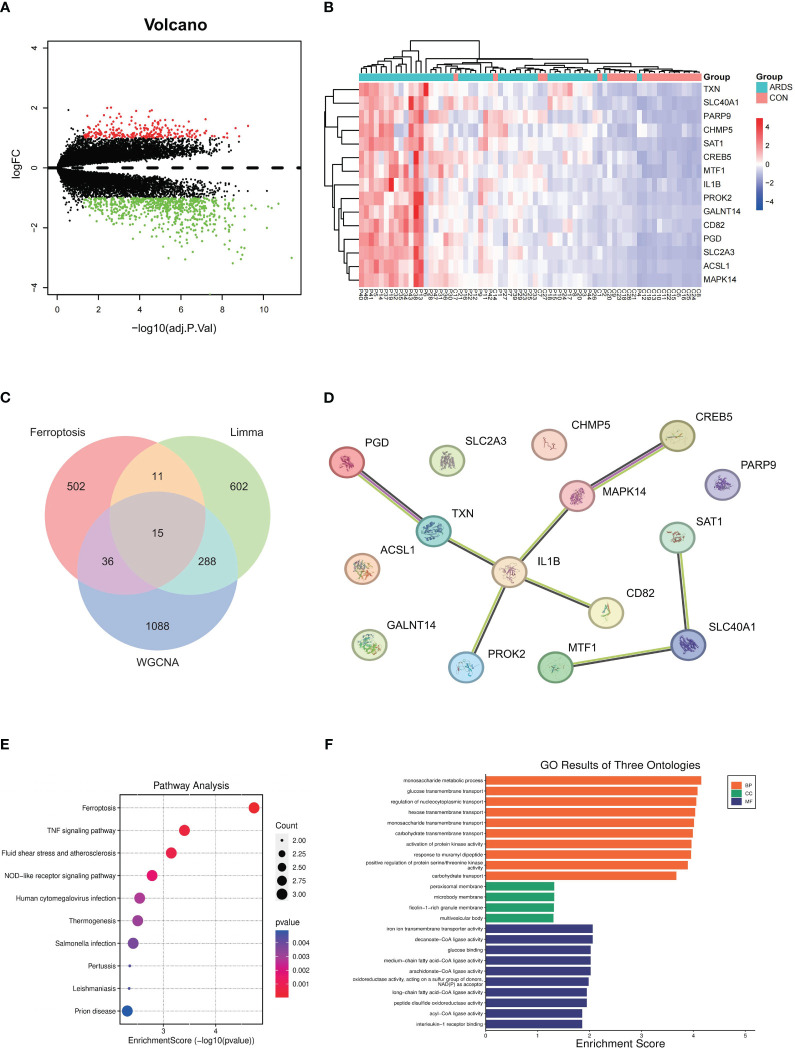
Hub genes identification and Functional enrichment analysis of GO/KEGG **(A)** differentially expressed genes between SARS-CoV-2 induced ARDS patients and non-critical patients, **(B)** expression levels of 15 different expressed genes in SARS-CoV-2 induced ARDS patients and non-critical patients, **(C)** Identification of the hub gene related to ferroptosis, **(D)** PPI network among 15 key genes, **(E)** pathway analysis outcomes, **(F)** GO results of three ontologies.

Next, we identified the most clinically relevant gene module based on its correlation with ARDS. The correlation between gene modules and clinical traits is depicted in the Module-trait relationships graph (**Figure 2B**). The inter-module correlations are depicted in Sup.1C. The brown and green-yellow modules are positively correlated with ARDS, with the former showing a correlation of 0.63 (p<0.05) and the latter 0.42 (p<0.05). Sup.1D display the GS-MM correlation for the brown and green-yellow modules, respectively, with a correlation coefficient of 0.52 in the brown module and 0.49 in the green-yellow module (p<0.05). Within the brown module, 1362 key genes were identified that met the screening criteria, and 64 in the green-yellow module.

### 3.3 Identification of differential expression genes

We combined the WGCNA method with differential gene expression analysis to further identify key genes in COVID-19-induced ARDS patients. According to the results from the limma R package, using the criteria of p-value ≤ 0.05 and |logFC| ≥ 1, a total of 916 differentially expressed genes (DEGs) were identified when comparing COVID-19 patients with moderate or severe ARDS to non-critical patients. Among these, 675 genes were upregulated and 241 genes were downregulated. These differentially expressed genes are depicted in a volcano plot (**Figure 2A**) where red represents up-regulated genes and green represents down-regulated genes. Heatmap (**Figure 2B**) was utilized to illustrate the expression profiles of 15 key genes across distinct patient groups.

### 3.4 Identification of the hub gene related to ferroptosis

To confirm the relationship between these genes and ferroptosis, we downloaded ferroptosis-related genes from the FerrDb database, including 369 drivers, 348 suppressors, 11 markers, and 117 unclassified genes. After removing duplicate genes, a total of 564 genes related to ferroptosis were obtained. After taking the intersection of these ferroptosis-related genes with the differential expression genes generated from limma and ARDS-related genes generated from WGCNA, we identified 15 hub genes (**Figure 2C**). The protein-protein interaction network diagram of these 15 key genes is shown in **Figure 2D**. SAT1, SLC40A1, and ACSL1 were enriched in the ferroptosis pathway, with MTF1 also being related to SLC40A1, yielding an interaction score of 0.426.

### 3.5 GO and KEGG pathway analysis

In our study, we explored the functions of genes associated with ferroptosis in ARDS and revealed their potential roles in biological processes (BP), cellular components (CC), and molecular functions (MF) through Gene Ontology (GO) analysis (**Supplementary Figures 2A–C**).

The BP analysis revealed that the differentially expressed genes (DEGs) associated with ferroptosis are predominantly enriched in various biological pathways related to carbohydrate metabolism. Additionally, these genes are implicated in the activation of protein kinase activity, response to peptidoglycan, and positive regulation of protein serine/threonine kinase activity, suggesting their potential role in regulating the signaling processes involved in ferroptosis and lung injury (**Figure 2E**).

The KEGG pathway enrichment analysis has demonstrated the roles of differentially expressed genes (DEGs) associated with several critical biological processes. Enriched pathways include ferroptosis and TNF signaling pathway. In addition, the enrichment of the NOD-like receptor signaling pathway suggests the involvement of innate immunity in ferroptosis (**Figure 2F**).

### 3.6 Immune infiltrating

**Figure 3:**
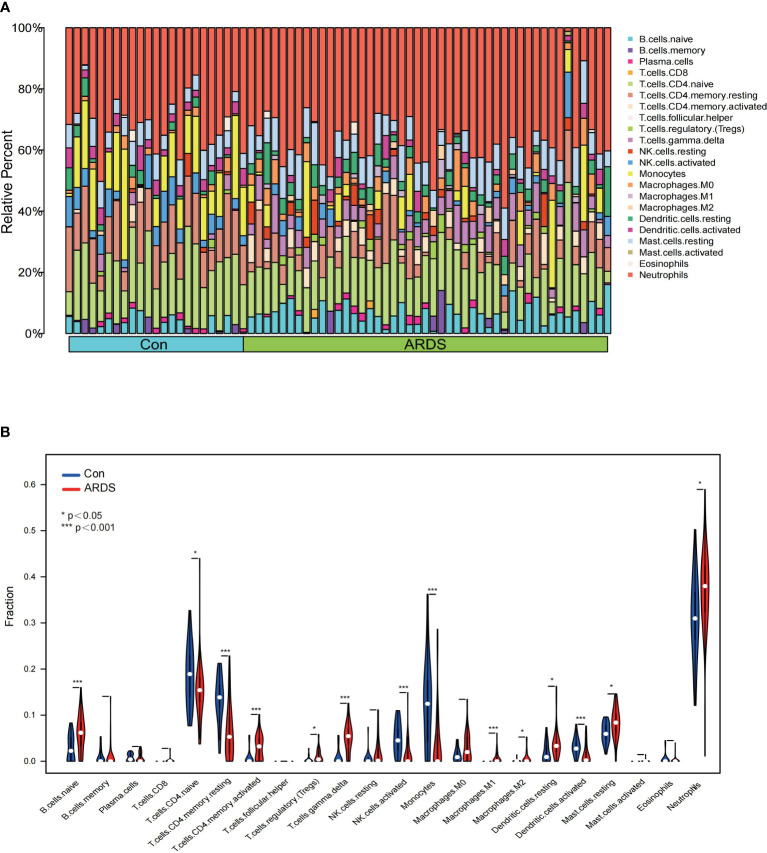
The result of immune infiltrating **(A)** proportions of 22 immune cell types across different individuals, **(B)** violin plot of proportions among 22 immune cell types between ARDS and non-critical patients.

**Figure 4:**
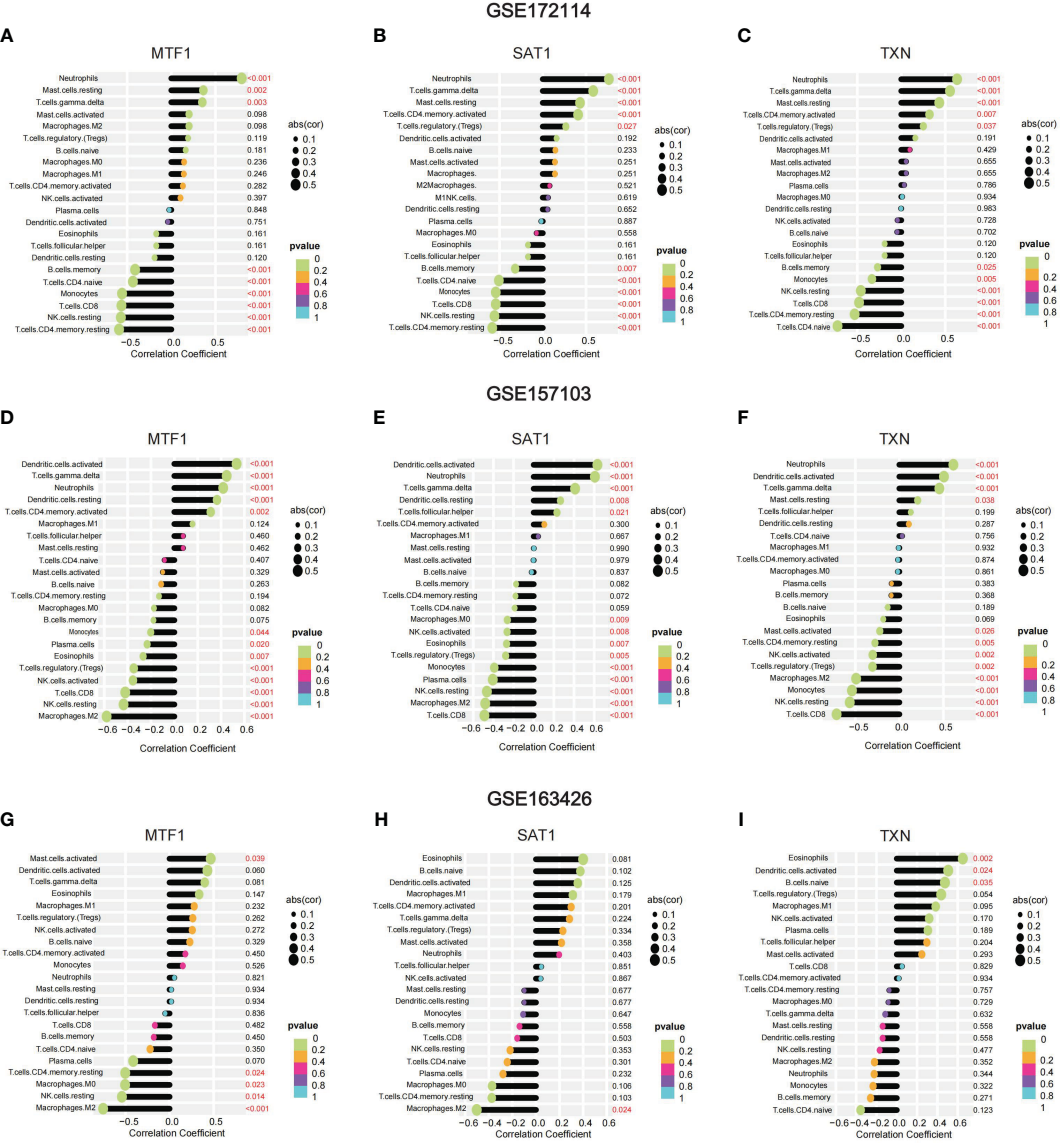
**(A–I)** Correlation between genes and 22 immune cells.

To confirm the distribution of immune cells in COVID-19-induced ARDS patients, we conducted immune infiltrating analysis on GSE172114. **Figure 3A** illustrates the proportions of 22 immune cell types across different individuals. According to the results of immune infiltration, compared to the non-critical group, ARDS patients showed a significant increase in the proportions of neutrophils, B cells Naive, T cells CD4 memory activated, and T cells gamma delta (p < 0.001), while the proportions of T cells CD4 Naive (p = 0.036), T cells CD4 memory resting, NK cells activated, monocytes, and activated dendritic cells (p < 0.001) were significantly decreased. The top five immune cells in ARDS patients were observed to be neutrophils, T cells CD4 Naive, B cells Naive, T cells gamma delta, and T cells CD4 memory resting (**Figure 3B**). Furthermore, by plotting the relationship between hub genes and immune cells, it was revealed that the proportion of neutrophils, mast cells resting, T cells gamma delta, exhibited a positive correlation with MTF1, SAT1, TXN, while B cell memory, T cells CD4 Naive, monocytes, T cells CD8, NK cells resting, T cells CD4 memory resting showed a negative correlation with them (**Figures 4A–C**). (Positive correlation: when the cell decreases, the gene expression level decreases. Negative correlation: when the cell decreases, the gene expression level increases) The correlation between genes and immune cells in GSM157103 and GSM163426 is as follows (**Figures 4D–I**).

### 3.7 Overview of hub gene expression in single-cell analysis

**Figure 5:**
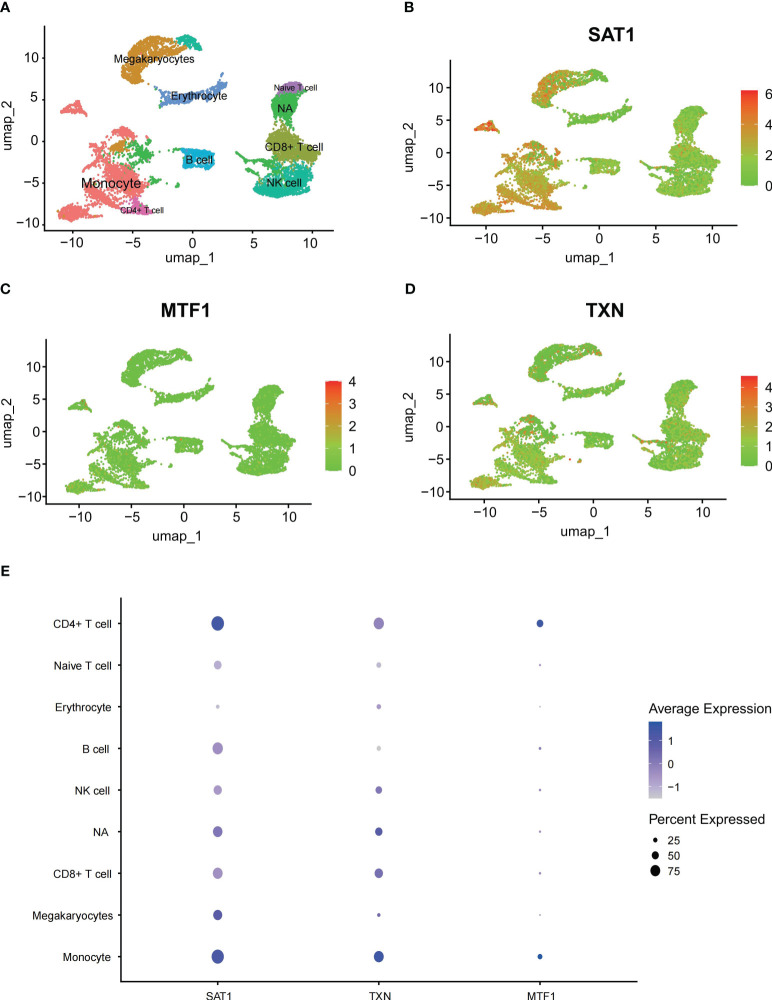
Single-cell analysis outcomes **(A)** UMAP plot all scRNA-seq cells color-coded by cell type, **(B–D)** scatter plots about SAT1, MTF1 and TXN, **(E)** bubble charts about SAT1, MTF1 and TXN.

This section aims to confirm the expression of genes in different immune cells of patients, corroborating the results of immune infiltration analysis. Using the Seurat package, we processed the data from GSE149689 with UMAP, identifying 26 distinct clusters. These clusters were classified into eight different cell types based on known cellular markers, including monocytes, platelet, CD8+ T cells, CD4+ T cells, Naive T cells, B cells, NK cells, and erythrocytes. Clusters 3, 14, 22, 23, 24, and 25 could not be definitively categorized into a specific cell type due to potential heterogeneity and were labeled as “NA” (**Figure 5A**). We illustrated the spatial distribution of ferroptosis-related genes using scatter plots (**Figures 5B–D**). Additionally, we plotted bubble charts to illustrate the changes in expression levels of key genes in immune cells such as monocytes and CD4+ cells (**Figure 5E**).

### 3.8 Validation of hub genes in GEO database and Vitro model

**Figure 6:**
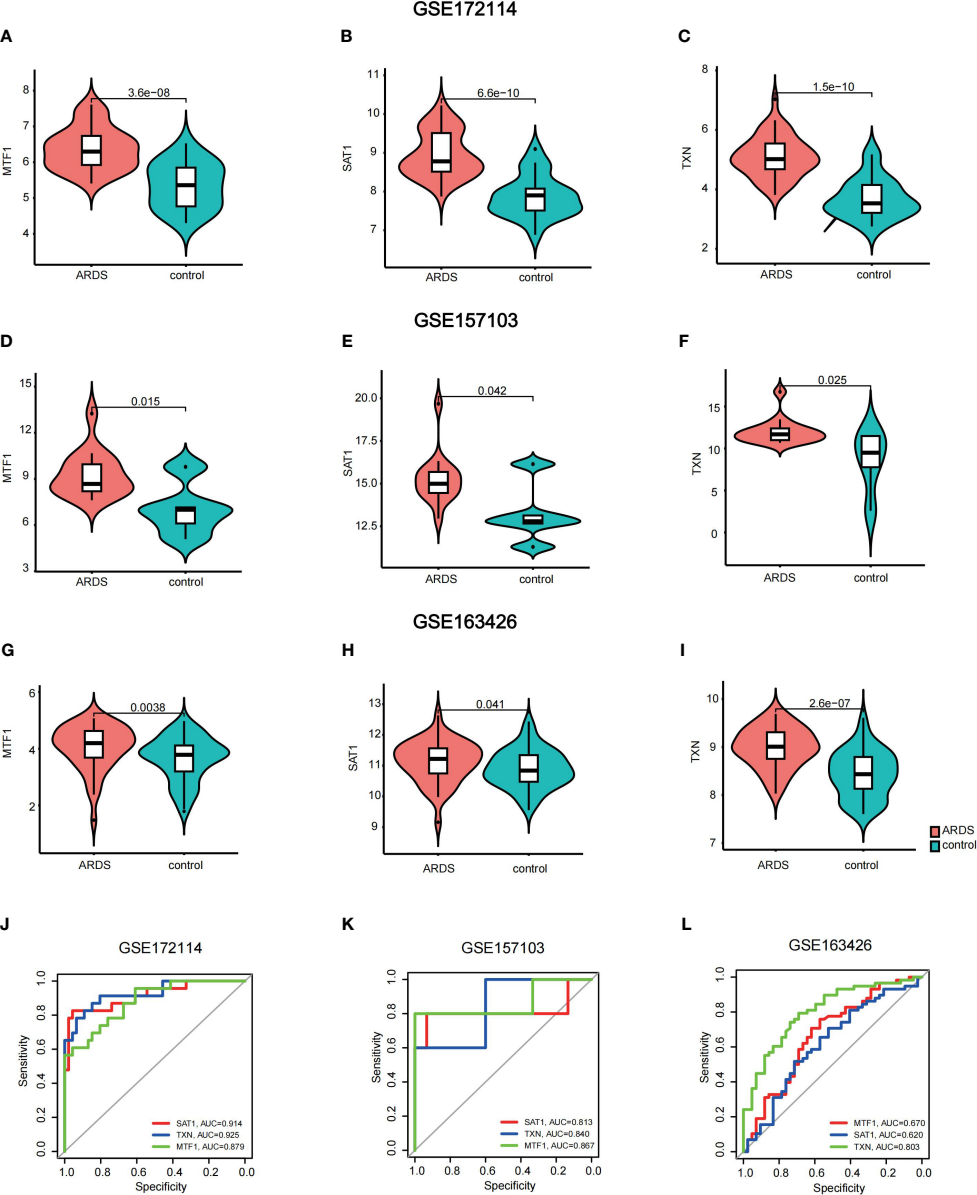
Validation of hub genes **(A–I)** violin plots of the expression of key genes in GSM172114, GSM157103, and GSM163426 **(J–L)** areas under the curve across GSM172114, GSM157103, and GSM163426.

**Figure 7:**
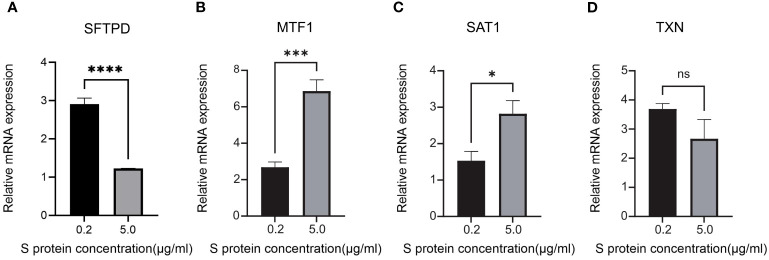
Relative mRNA expression levels of SFTPD, MTF1, SAT1 and TXN in BEAS-2B cells of 0.2 g/ml Sproteinconcentration and 5.0 ug/ml S protein concentration **(A)** SFTPD, **(B)** MTF1, **(C)** SAT1, **(D)** TXN. *P<0.05, ***P<0.001, ****p<0.0001.

To validate the expression of key genes in COVID-19-induced ARDS, GSE157103 was employed. GSE163426 was selected to exclude the impact of mechanical ventilation factors on gene expression dynamics during the progression of ARDS. According to **Figures 6A–I**, we found that in both total blood and tracheal aspirate samples, MTF1, SAT1, and TXN exhibited a statistically significant upregulation in COVID-19-induced ARDS. After that, the diagnosis value of the above three hub genes was verified by using the ROC curve. In **Figures 6J–L**, three hub genes exhibit significant areas under the curve across GSM172114, GSM157103, and GSM163426. Then, we set up BEAS-2B vitro model to further validate key gene expression. We measured the mRNA relative expression of the SFTPD gene, which demonstrated the success of the BEAS-2B cell injury model. The mRNA relative expression content of SFTPD and other genes was as shown in **Figure 7A**, and the relative expression of SFTPD mRNA decreased with the increase of S protein concentration. The expression levels and differences of SAT1, MTF1, and TXN in the groups stimulated with 0.2 µg/ml and 5.0 µg/ml S protein are shown in **Figures 7B–D**. Compared to the group stimulated with 0.2 µg/ml S protein, the group stimulated with 5.0 µg/ml S protein exhibited increased expression levels of SAT1 and MTF1, which is consistent with our predicted expression trends.

## 4 Discussion

Ferroptosis is a unique iron-dependent, non-apoptotic programmed form of cell death, characterized by the production and accumulation of reactive oxygen species (ROS) leading to lipid peroxidation (13). Ferroptosis is mainly manifested by an increase in mitochondrial membrane density and mitochondrial shrinkage. Recent studies have shown that ferroptosis is closely associated with various respiratory diseases, such as asthma (34), chronic bronchitis (35), lung cancer (36), etc. Experimental evidence has demonstrated that ferroptosis constitutes a significant mechanism involved in the pathogenesis of ARDS, which can influence inflammation and oxidative stress, thereby achieving the goal of alleviating or improving ARDS (19, 21, 22, 37). Consequently, novel therapeutic strategies for ARDS are increasingly emphasizing the inhibition of ferroptosis onset (38).

However, the mechanism by which ferroptosis occurs in viral-induced ARDS, such as SARS-CoV-2 and influenza viruses, remains unclear. This experiment hopes to use bioinformatics methods to identify the key genes and pathways that are involved in ferroptosis-induced ARDS, with the expectation of discovering key genes and targets to provide a new foundation for the treatment of ARDS.

In this study, the initial screening was conducted using the Weighted Gene Co-expression Network Analysis (WGCNA) method and differential gene expression analysis. A total of 15 ferroptosis-related genes with significant differences in the occurrence of ARDS induced by COVID-19 were identified. To refine the precision of our research objectives, we delved into the known functions of these key genes using the GeneCards website (https://www.genecards.org/cgi-bin/carddisp.pl). The association strength with ARDS among these 15 genes was observed through enrichment analysis results and Protein-Protein Interaction (PPI) results. Ultimately, MTF1, SAT1, and TXN were selected as the hub genes of interest.

MTF1, or metal regulatory transcription factor 1, is a conserved metal-binding transcription factor found in eukaryotes. When cellular metal levels exceed normal thresholds, MTF1 responds by activating protective mechanisms against oxidative and stress-induced damage. The enhanced expression of the MTF1 gene is responsible for regulating the expression of Ferritin/FPN1 and providing protection against ferroptosis (39). MTF1 is highly expressed in human bone marrow and lymphoid tissues, indicating that it may play a crucial role in the proliferation and differentiation of immune cells in COVID-19 induced ARDS patients. Furthermore, recent studies propose that MTF1 may serve as a predictive biomarker for COVID-19 (40). In this study, we observed a significantly elevated expression of MTF1 in ARDS patients compared to the control group, and a similar increase in MTF1 expression was also noted in the validation group. We speculate that this heightened expression is a response to iron overload occurring during the induction of ferroptosis in the ARDS process. Consequently, in response to intracellular iron overload, the upregulation of MTF1 expression enhances the expression of iron transport proteins, facilitating the efflux of excess iron from cells and thereby mitigating the occurrence of ferroptosis. This hypothesis aligns with the findings reported by D. M. Ward et al. (41).

SAT1, also recognized as spermidine/spermine N1-acetyltransferase 1, represents a crucial polyamine catabolic enzyme. In physiological conditions, the expression level of the SAT1 gene is relatively low, but they undergo elevation in response to various stimuli such as drugs, stress, and inflammation (42, 43). SAT1 exhibits high levels of RNA and protein expression in the human respiratory system, with significant distribution in lung epithelial cells and type II alveolar cells. This provides important evidence linking ferroptosis to ARDS caused by COVID-19. Ou et al. demonstrated that SAT1 is involved in regulating p53-mediated reactive oxygen species (ROS) responses and ferroptosis, leading to the accumulation of lipid peroxides and harmful effects on normal cells, even resulting in cell death (44). In this experiment, it was found that SAT1 expression increased in the ARDS group compared to the control group, and this conclusion was also validated in the verification group. This suggests that in patients with COVID-19-induced ARDS, SAT1 is an essential factor contributing to ferroptosis.

TXN, Thioredoxin in full, encodes a protein that functions as a homodimer participating in numerous redox reactions, making it a crucial regulator of the redox balance. TXN exhibits diverse biological functions, including the repair of DNA damage (45), activation of transcription factors (46), inhibition of apoptotic signaling pathways, and elimination of reactive oxygen species (ROS). It enhances cellular resistance to oxidative stress and promotes cell proliferation. TXN is widely expressed in various tissues and organs throughout the body, suggesting that changes in TXN expression may be related to the systemic inflammatory response caused by ARDS. Literature indicates that downregulation of TXN gene expression can inhibit B regulatory cell activity, promote the differentiation of pro-inflammatory B cells, and induce systemic inflammation (47). Qian Li et al. (48)validated, through mouse experiments, that increased expression of TXN can suppress lung tissue damage in diseased mice, leading to improved mouse survival rates.

Across various datasets, it was observed that the expression of TXN in the ARDS group is significantly higher than in the control group. This indicates that during the progression to ARDS in patients, TXN is consistently upregulated to inhibit inflammation development and alleviate the severity of ARDS. TXN holds promise as a therapeutic target for treating and preventing the progression of the disease to the ARDS.

After establishing the final key genes, we conducted validation and analysis on these key genes. Utilizing violin plots, we observed that the alteration trends in the expression of hub genes within the validation dataset mirrored those in GSM172114, confirming the heightened expression of SAT1, MTF1, and TXN in the COVID-19-induced ARDS group. Using qRT-PCR, we found that under S protein stimulation, the expression levels of MTF1 and SAT1 genes increased with higher concentrations of S protein in BEAS-2B vitro model, which aligns with our predictions. In the future, we plan to include clinical sample experiments to obtain more accurate validation. Additionally, hen MTF1, SAT1, and TXN were used individually as diagnostic indicators, MTF1, SAT1, and TXN demonstrated an area under the curve (AUC) surpassing 0.5. Particularly, TXN exhibited an AUC greater than 0.800 in all three datasets, indicating favorable sensitivity and specificity as a diagnostic indicator.

In the lollipop plots of GSE172114 and GSE157103, a significant positive correlation was observed between neutrophils and gamma delta T cells with the genes MTF1, SAT1, and TXN. However, in GSE163426, no evident correlation was observed between hub genes and various immune cells. Elevated neutrophil levels are commonly observed in inflammatory, tumor, and stress conditions. Kun et al. found that regulating neutrophil phenotype can alleviate acute inflammation and diffuse alveolar injury in the lungs (49).

Gamma delta T cells, as an independent subset of T cells, tend to accumulate in lung tissues. Most studies have focused on the role of gamma delta T cells in different allergic response models in the lungs, but the conclusions are contradictory (50–52). Born et al. discovered the protective role of gamma delta T cells against pulmonary fibrosis (53). However, further research is still needed to fully understand the functions of gamma delta T cells in lung tissues.

According to the single-cell sequencing results from GSE148689, it is evident that the expression of hub genes in monocytes is significantly upregulated in ARDS patients. In macrophages derived from monocytes, knocking down MTF1 can lead to a decrease in pathogen clearance, delaying disease recovery (54). Other scRNA sequencing results also support the conclusion that SAT1 and TXN are elevated in severe COVID-19 patients (55, 56).

This study has several limitations. Firstly, the validation group employed bioinformatics analysis methods for validation without experimental confirmation. Secondly, while the majority of expression trends in the validation group did align with GSE172114, the remaining genes did not display differential expression, thereby decrease the overall reproducibility of the experimental data.

## 5 Conclusion

We have identified MTF1, SAT1, and TXN genes as potential key genes involved in ferroptosis induced by SARS-CoV-2 associated ARDS. These findings present novel targets for immune therapy and targeted treatment of ARDS. Further research is required to elucidate the specific mechanisms through which ferroptosis triggers ARDS in the context of COVID-19.

## Data availability statement

The original contributions presented in the study are included in the article/**Supplementary Material**. Further inquiries can be directed to the corresponding author.

## Author contributions

YL: Conceptualization, Data curation, Formal analysis, Funding acquisition, Investigation, Methodology, Project administration, Resources, Software, Supervision, Validation, Visualization, Writing – original draft, Writing – review & editing. LT: Conceptualization, Data curation, Formal analysis, Investigation, Methodology, Project administration, Resources, Software, Supervision, Validation, Visualization, Writing – original draft, Writing – review & editing. FW: Conceptualization, Funding acquisition, Methodology, Project administration, Supervision, Writing – review & editing. CG: Data curation, Funding acquisition, Methodology, Project administration, Software, Supervision, Validation, Writing – review & editing. QY: Formal analysis, Investigation, Methodology, Validation, Visualization, Writing – review & editing. LL: Data curation, Project administration, Software, Supervision, Validation, Writing – review & editing. JW: Supervision, Validation, Writing – review & editing, Data curation, Software. QT: Data curation, Investigation, Software, Writing – review & editing. MQ: Conceptualization, Formal analysis, Funding acquisition, Methodology, Project administration, Supervision, Writing – original draft, Writing – review & editing.
