# Supplementary material for: Hub genes identification and validation of ferroptosis in SARS-CoV-2 induced ARDS: perspective from transcriptome analysis
Source: Front Immunol. 2024 Aug 7;15:1407924. doi: 10.3389/fimmu.2024.1407924 (PMC11335500; doi:10.3389/fimmu.2024.1407924)
Supplement: Supplementary file 1 [file Table_1.docx]

| **Gene** | **Primer sequences (5' to 3')** | |
| --- | --- | --- |
| **SAT1** | **Forward** | **CTTCTTGGTAGCAGAATGGAATGAAC** |
| **SAT1** | **Reverse** | **CTCCTTGTCGATCTTGAACAGTCTC** |
| **MTF1** | **Forward** | **CAGCCTGGACGAATGATGCC** |
| **MTF1** | **Reverse** | **TCAGTTGTCCTTGGTTATTGATGGG** |
| **TXN** | **Forward** | **ATGAAAGAAAGGCTTGATCATTTTGC** |
| **TXN** | **Reverse** | **TAAACTTGTAGTAGTTGACTTCTCAGC** |
| **SFTPD** | **Forward** | **ACAGGCTGGTGGACAGTTGG** |
| **SFTPD** | **Reverse** | **GTCTTGGAATCAGTCATGCTCAGG** |
| **GAPDH** | **Forward** | **GTCTCCTCTGACTTCAACAGCG** |
| **GAPDH** | **Reverse** | **ACCACCCTGTTGCTGTAGCCAA** |

**Supplementary Table 1. Primer sequences**
