# Supplementary material for: Hub genes identification and validation of ferroptosis in SARS-CoV-2 induced ARDS: perspective from transcriptome analysis
Source: Front Immunol. 2024 Aug 7;15:1407924. doi: 10.3389/fimmu.2024.1407924 (PMC11335500; doi:10.3389/fimmu.2024.1407924)
Supplement: Supplementary file 2 [file Image_1.pdf]

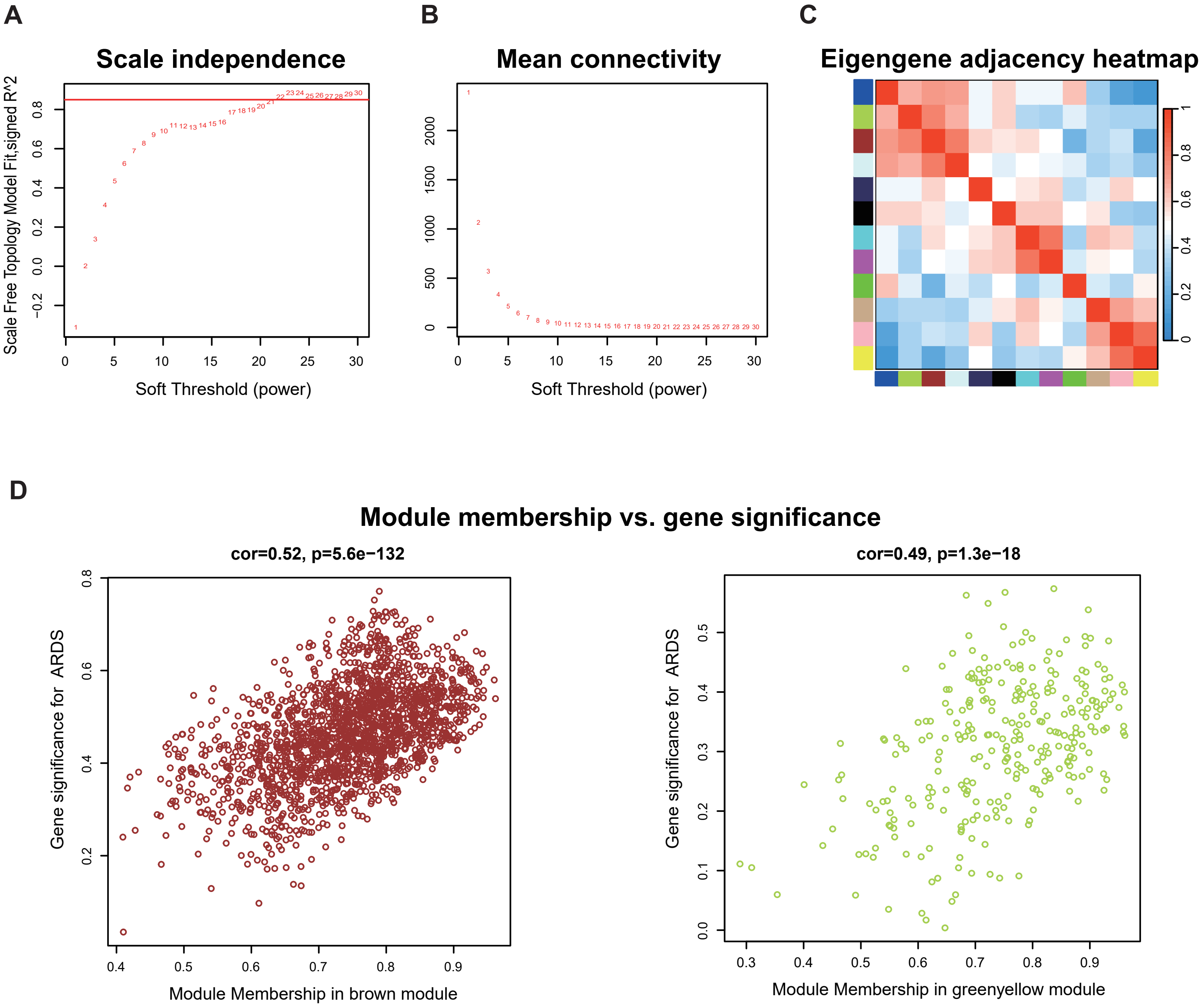
A
Scale independence
Scale Free Topology Model Fit,signed R^2
Soft Threshold (power)
B
Mean connectivity
Soft Threshold (power)
C
Eigengene adjacency heatmap
D
Module membership vs. gene significance
cor=0.52, p=5.6e−132
Gene significance for ARDS
Module Membership in brown module
cor=0.49, p=1.3e−18
Gene significance for ARDS
Module Membership in greenyellow module
