# Supplementary figures and images for: Hub genes identification and validation of ferroptosis in SARS-CoV-2 induced ARDS: perspective from transcriptome analysis
Source: Front Immunol. 2024 Aug 7;15:1407924. doi: 10.3389/fimmu.2024.1407924 (PMC11335500; doi:10.3389/fimmu.2024.1407924)

A

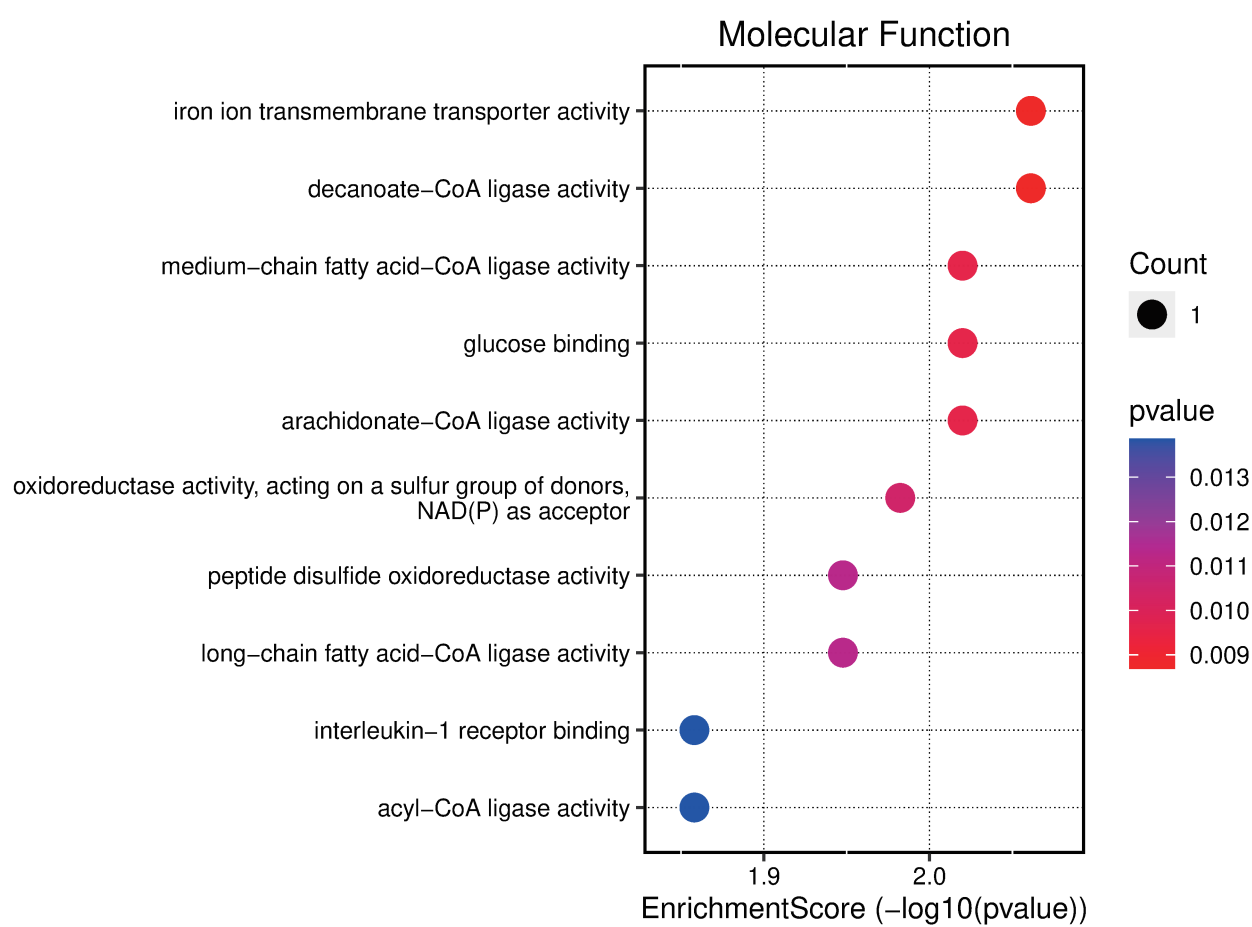

B

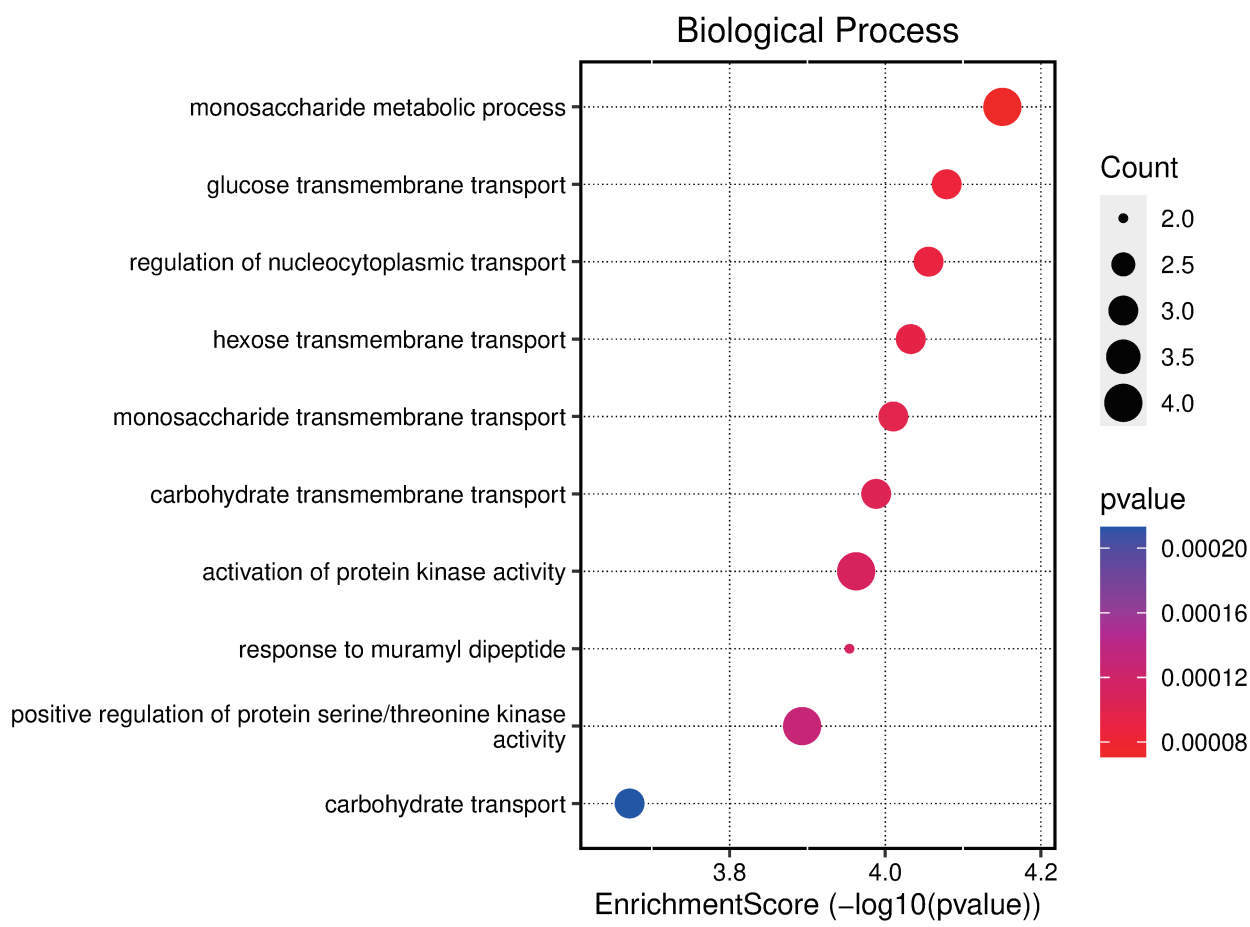

C

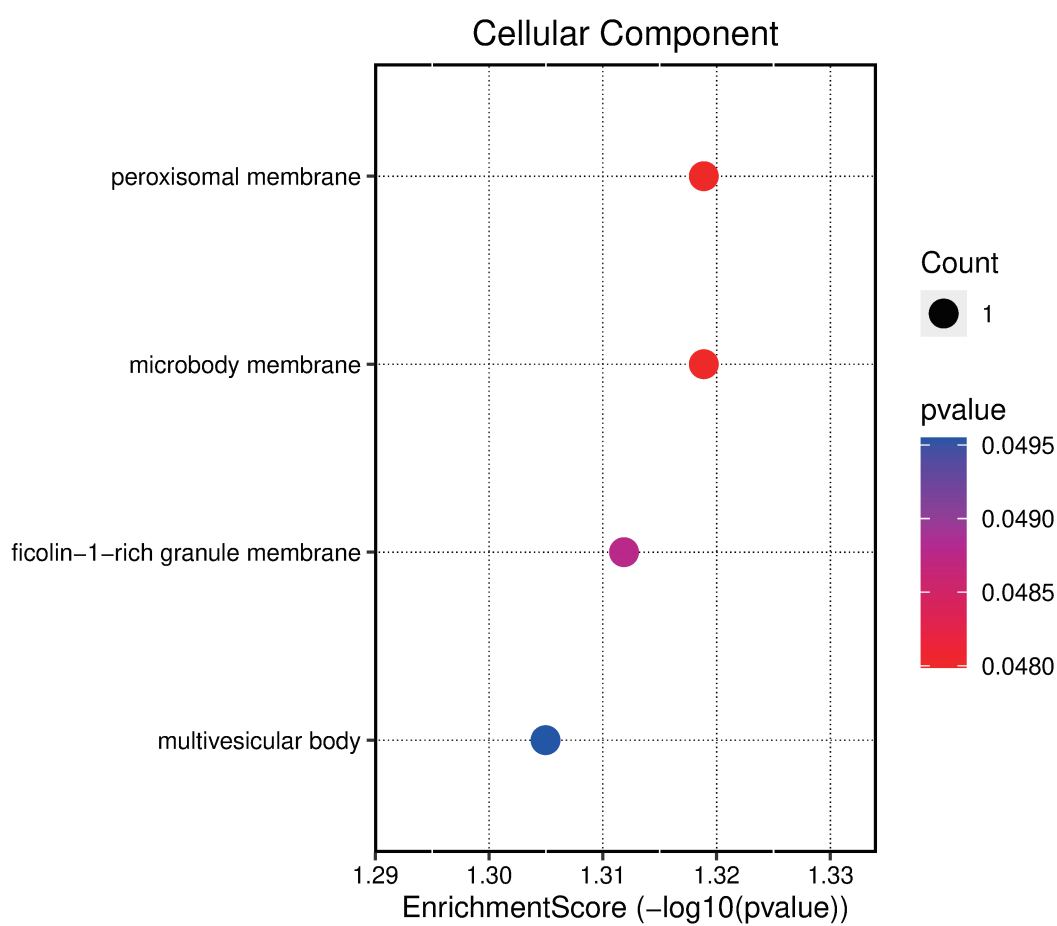

Supplement: Supplementary file 3 [file Image_2.pdf]
